# S100A4^+^ Macrophages Are Necessary for Pulmonary Fibrosis by Activating Lung Fibroblasts

**DOI:** 10.3389/fimmu.2018.01776

**Published:** 2018-08-06

**Authors:** Yanan Li, Jing Bao, Yangyang Bian, Ulrike Erben, Peigang Wang, Kun Song, Shuangqing Liu, Zhenzhen Li, Zhancheng Gao, Zhihai Qin

**Affiliations:** ^1^Key Laboratory of Protein and Peptide Pharmaceuticals, CAS Center for Excellence in Biomacromolecules, Chinese Academy of Sciences-University of Tokyo Joint Laboratory of Structural Virology and Immunology, Institute of Biophysics, Chinese Academy of Sciences, Beijing, China; ^2^University of Chinese Academy of Sciences, Beijing, China; ^3^Department of Respiratory and Critical Care Medicine, Peking University People’s Hospital, Beijing, China; ^4^Medical Research Center, The First Affiliated Hospital of Zhengzhou University, Zhengzhou, China

**Keywords:** S100A4, macrophages, fibroblasts, pulmonary fibrosis, sphingosine-1-phosphate

## Abstract

S100A4, a calcium-binding protein, can promote pulmonary fibrosis *via* fibroblast activation. Due partly to its various cellular origins, the exact role of S100A4 in the development of lung fibrosis remains elusive. Here, we show that in the bronchoalveolar lavage fluid, numbers of S100A4^+^ macrophages correlated well with S100A4 protein levels and occurrence of idiopathic pulmonary fibrosis (IPF) in patients. A mouse model of bleomycin-induced pulmonary fibrosis demonstrated S100A4^+^ macrophages as main source for extracellular S100A4 in the inflammatory phase. *In vitro* studies revealed that extracellular S100A4 could activate both mouse and human lung fibroblasts by upregulation of α-SMA and type I collagen, during which sphingosine-1-phosphate (S1P) increased. Inhibiting the S1P receptor subtypes S1P_1_/S1P_3_ abrogated fibroblast activation. Accordingly, absence or neutralization of S100A4 significantly attenuated bleomycin-induced lung fibrosis *in vivo*. Importantly, adoptive transfer of S100A4^+^ but not of S100A4^−^ macrophages installed experimental lung injury in S100A4^−/−^ mice that were otherwise not sensitive to fibrosis induction. Taken together, S100A4 released by macrophages promotes pulmonary fibrosis through activation of lung fibroblasts which is associated with S1P. This suggests that extracellular S100A4 or S100A4^+^ macrophages within the lung as promising targets for early clinical diagnosis or therapy of IPF.

## Introduction

Idiopathic pulmonary fibrosis (IPF) is a progressive lung-scarring disorder with difficult early diagnosis (1) and diagnosing IPF requires multidisciplinary clinical approaches (2). Serological evaluation for pulmonary fibrosis is recommended but the signs are so far not specific enough (3). Late diagnosis combined with unresponsiveness to available therapy options (4, 5) leads to high mortality from IPF (6). Therefore, biomarkers accessible from bronchoalveolar lavage fluid (BALF) or peripheral blood (7, 8) hold the potential for early diagnosis and improvement of therapeutic outcome.

Inflammation is crucial for the onset and development of IPF. Innate and adaptive immune mechanisms contribute to the final stereotypical pathways leading to severe pulmonary fibrosis (9). Monocytes that transform into activated macrophages play an important role in promoting this process. Although exact mechanisms are still not clear, early inflammatory responses mediated by transforming growth factor-β, tumor necrosis factor-α, interleukin (IL)-1, IL-6, or CC-chemokine ligand-2 help to drive inflammatory responses (10, 11). By directly activating fibroblasts to proliferate and to become myofibroblasts, they also trigger the accumulation of extracellular matrix components (9, 12–14). However, it is still not clear, which cytokine can be used as the target for an effective IPF treatment.

S100A4 is a member of the family of small Ca^2+^ binding protein. As an intracellular molecule, it regulates cellular biological functions, such as cell mobility, proliferation, or metastasis (15–17). In addition to this, S100A4 can also be actively released by different cells (18). Extracellular S100A4 acts *via* promiscuous surface receptors like receptor for advanced glycation end products (RAGE), annexin II, heparan sulfate proteoglycans, or toll-like receptor 4 (19–22). Recently, S100A4 from fibroblasts is shown to promote fibrosis in different tissues, including liver, myocardium, kidney, and lung (23, 24). Interestingly, S100A4 is also secreted by immune cells, such as macrophages (18, 25, 26). In fact, in pulmonary fibrosis, a subpopulation of S100A4^+^ cells co-expresses CD45, a hematopoietic cell marker (27). The role of S100A4 in these cells during induction and progress of pulmonary fibrosis and IPF is elusive.

Here, we observed that S100A4 protein levels and the amount of S100A4^+^ macrophages correlated with the occurrence of IPF in patients. Increased numbers of S100A4^+^ macrophages were also found in lung tissues of bleomycin-treated mice during the development of pulmonary fibrosis. S100A4 deficiency (S100A4^−/−^) or interestingly, blocking of S100A4 using a neutralizing antibody reduced fibrosis in the animal model. Proving the causal connection, adoptive transfer of S100A4^+^ macrophages to bleomycin-treated syngeneic S100A4^−/−^ mice augmented pulmonary fibrosis. Strong activation of lung fibroblasts by exogenous S100A4 involved an altered conversion kinetics of sphingosine-1-phosphate (S1P) to sphingosine (SPH). Therefore, macrophage-derived S100A4 is necessary and sufficient for the induction of lung fibrosis in mice.

## Materials and Methods

### Human Samples

Cell-free BALF samples (1 ml) and slides with cellular smears of patients with IPF (*n* = 17) and or non-IPF lung diseases (*n* = 25) were obtained from the Respiratory and Critical Care Medicine department, Peking University People’s Hospital (Beijing, China). The latter group included patients with pneumonia (*n* = 8), tuberculosis (*n* = 3), hypersensitivity pneumonitis (*n* = 2), and non-specified non-infectious lung disease (*n* = 12). Patient information is summarized in Table S1 in Supplementary Material. Surgical lung tissue samples of lung carcinoma patients (*n* = 2) were provided by the Medical Research Center of the First Affiliated Hospital of Zhengzhou University (Zhengzhou, China). Primary human lung fibroblasts from tissues adjacent to lung carcinoma were isolated by enzymatic digestion and cultured for about five passages. The study was approved by the Ethics Committee at Peking University People’s Hospital.

### Cell Lines

MRC-5 human lung fetal fibroblast cells and murine RAW264.7 macrophages were purchased from China Infrastructure of Cell Line Resources (Beijing, China). The cells were passaged twice weekly in Dulbecco’s Modified Eagle Medium containing 10% fetal bovine serum, 100 U/ml penicillin, and 100 µg/ml streptomycin. They were incubated at 37°C in a humidified atmosphere of 5% CO_2_ and 95% air and monthly checked for mycoplasma contamination.

### Mouse Strains

C57BL/6 mice (WT) were purchased from Vital River (Beijing, China). Heterozygous B6.Cg-Tg (S100a4-EGFP) M1Egn (S100A4^+/+GFP^) and homozygous B6.129S6-S100a4^tm1Egn^ (S100A4^−/−^) transgenic mice were purchased from Jackson Laboratory (Bar Harbor, ME, USA). All mice were bred under specific pathogen-free conditions in the animal facilities at the Institute of Biophysics, Chinese Academy of Sciences (Beijing, China). All animal studies were performed with sex- and age-matched mice after being approved by the Institutional Laboratory Animal Care and Use Committee.

### Mouse Model of Pulmonary Fibrosis

Mice were treated once with intratracheal instillation of 5 mg/kg bleomycin (Nippon Kayaku, Kyoto, Japan) after anesthesia by intraperitoneal injection of 5% chloral hydrate as described previously (28). Mice were sacrificed, and tissues were harvested up to 4 weeks after bleomycin administration. All mice were grouped randomly for experiments.

### Recombinant S100A4 and S100A4-Specific Monoclonal Antibody

Purified recombinant murine S100A4 protein and a neutralizing monoclonal mouse-derived antibody specific for murine and human S100A4 (clone 3B11; anti-S100A4) were produced as previously described (29). To be used in the experiments, endotoxin concentrations in all preparations were <1 EU/ml as tested by endotoxin assay kit (Genscript, Nanjing, China).

### *In Vitro* Treatment of Lung Fibroblasts

Cells from cell lines or freshly isolated cells (2 × 10^5^) were cultured in 6-well cell culture dishes (Corning, NY, USA) and RPMI-1640 supplemented with 10% fetal bovine serum, 100 U/ml penicillin, and 100 µg/ml streptomycin. Recombinant exogenous S100A4 was added as indicated. If applicable, S100A4 was preincubated with a twofold molar excess of anti-S100A4 for 30 min at 4°C. S1P function was altered by using the S1P_1_/S1P_3_ antagonist VPC23019 (Cayman, Ann Arbor, MI, USA), S1P_2_ antagonist JTE013 (Selleck, Shanghai, China), transforming growth factor-β (TGF-β) was purchased from R&D Systems (Minneapolis, MN, USA).

### Flow Cytometry Analysis

Single cell suspensions of peripheral blood or prepared from lung tissue were stained with the following directly labeled mouse-specific monoclonal antibodies: anti-CD11b, anti-F4/80, anti-CD4, anti-CD8, anti-B220, and anti-Ly6C/6G. Final concentrations were 200 ng/ml; detailed information is given in Table S2 in Supplementary Material. Stained cells were collected using a FACSCalibur device (BD Biosciences, San Diego, CA, USA) and analyzed by the FlowJo software (TreeStar, Ashland, OR, USA).

### Adoptive Transfer of CD11b^+^S100A4^+^/S100A4^−^ Cells

S100A4^+/+GFP^ and S100A4^−/−^ mice were treated with bleomycin as described above. After 1 week, collected spleen cells from S100A4^+/+GFP^ mice were stained for CD11b and sorted on a FACSAria IIIu device (BD Biosciences). GFP served as reporter for S100A4 expression. To be used in the transfer model, purity of S100A4^+^CD11b^+^ and S100A4^−^CD11b^+^ cells had to be about 95% as determined by flow cytometry. CD11b^+^ cells spleen sorted according to the S100A4 expression (2 × 10^6^) were injected into the tail vein of bleomycin-treated S100A4^−/−^ mice that were studied after another week.

### Western Blot Analysis

Cells (1 × 10^5^) were harvested and lysed by RIPA buffer as described earlier (18). Cell extracts were collected and stored at −80°C. Proteins were separated by homogeneous SDS-PAGE and transferred to a nitrocellulose membrane (pore size 0.45 µm; Merck Millipore, Darmstadt, Germany). Table S3 in Supplementary Material summarized information about primary antibodies: anti-α-SMA, anti-α2 chain of type-1 collagen (COL1A2), anti-p-ERK, anti-p-AKT, anti-p-p65, anti-ERK, anti-AKT, and anti-p65. Primary antibody binding was visualized by chemiluminescence using horseradish peroxidase-conjugated goat anti-mouse (1:5,000) or goat anti-rabbit IgG secondary antibodies (1:3,000; both Sigma-Aldrich, St. Louis, MO, USA).

### Immunohistochemistry and Immunofluorescence for Tissues

Sections of frozen or formalin-fixed paraffin embedded murine lung tissue samples (5 µm) were stained with H&E for histological overview. Sirius Red (0.1%) and Fast Green (0.1%; both Beyotime, Shanghai, China) in picric acid were used to mark intercellular collagen deposition. Stained sections were analyzed by standard brightfield microscopy (CKX41, Olympus, Tokyo, Japan). For immunofluorescence *in situ*, sections were incubated with the following mouse specific raised in rat or rabbit: anti-α-SMA, anti-CD11b, anti-F4/80, and anti-S100A4; details are given in Table S3 in Supplementary Material. Specific antibody binding was visualized using AF488-conjugated goat anti-rabbit (1:200) or AF555-conjugated goat anti-rat (1:200; both BioLegend, San Diego, CA, USA) secondary antibodies. Nuclei were counterstained with 4′,6-diamidino-2-phenylindole (DAPI; Sigma-Aldrich). Stained tissue sections were analyzed using a standard fluorescence microscope (DP71; Olympus).

The extent of tissue areas stained by Sirius Red or for α-SMA was estimated and quantified in relation to the total tissue area using the Image J software (Media Cybernetics, Bethesda, MD, USA).

### Enzyme-Linked Immunosorbent Assay (ELISA)

S100A4 protein levels in human BALF were detected by ELISA. In brief, 96-well plates (Corning, Tewksbury, MA, USA) were coated with anti-S100A4 (1 µg/ml; clone 3B11) (Purified by Cwbio, Beijing, China) in carbonate solution (pH 9.6). BALF samples, diluted 1:5 with phosphate-buffered saline (pH 7.4) containing 3% bovine serum albumin (Thermo Fisher Scientific, Waltham, MA, USA) were incubated for 1 h at 37°C. Captured antigen was detected with rabbit polyclonal anti-S100A4 (1:1,000; Abcam, Cambridge, UK). The complex was visualized by horseradish peroxidase-conjugated anti-rabbit IgG (1:3,000; Sigma-Aldrich, St. Louis, MO, USA) and TMB substrate (Cwbio). Absorbance was detected using a microplate reader (EPOCH12; Instruments, Winooski, VT, USA). Serum concentrations were calculated from a standard curve of recombinant human S100A4 (R&D Systems, Minneapolis, MN, USA) in a concentration range of 12.5–1,000 ng/ml.

Blood was collected from eyeball venous plexus of mice, and the serum stored at 4°C. S1P or SPH was detected by ELISA kits (Enzyme-Linked Biotechnology, Shanghai, China) according to the manufacturer’s instructions.

### Quantitative Real-Time PCR

Total RNA was extracted from cells (1 × 10^5^) using TRIzol (Tiangen, Beijing, China), and cDNA was synthesized using EasyScript First-Strand cDNA Synthesis SuperMix (TransGen, Beijing, China). SYBR Green (TransGen). Quantitative real-time PCR was performed using a Rotor-GeneTM6000 (Corbett, Australia) and the following primer pairs (forward/reverse; 5′–3′): mouse α-Sma (AAGCCCAGCCAGTCGCTGTCA/GAAGCCGGCCTTACAGAGCCC) (18), mouse Col1a2 (GCTCCTCTTAGGGGCCACT/ATTGGGGACCCTTAGGCCAT) (18), mouse glyceraldehyde-3-phosphate dehydrogenase (Gapdh; TGGCCTTCCGTGTTCCTAC/GAGTTGCTGTTGAAGTCGCA) (18), mouse S1pr1 (ATGGTGTCCACTAGCATCCC/CGATGTTCAACTTGCCTGTGTAG Primerbank ID 21687214a1), mouse S1pr2 (ATGGGCGGCTTATACTCAGAG/GCGCAGCACAAGATGATGAT Primerbank ID 4324651a1), mouse S1pr3 (ACTCTCCGGGAACATTACGAT/CAAGACGATGAAGCTACAGGTG Primerbank ID 6753716a1), mouse S1pr4 (CCAAGACCAGCCGTGTGTAT/CCACTCTAAAGATGGCCCCG, NM_010102.2), S1pr5 (CTAGGGCACACGACCGGA/GTCTCCTGTAACCGGCACTC, NM_053190.2), human *ASMA* (TTCATCGGGATGGAGTCTGCTGG/TCGGTCGGCAATGCCAGGGT) (18), human *COL1A2* (TGATGGGATTCCCTGGACCT/GGGCCTTGTTCACCTCTCTC-3′) (18), and human *GAPDH* (TGTTGCCATCAATGACCCCTT/CTCCACGACGTACTCAGCG) (18). Individual mRNA amounts were quantified from Ct values in comparison with standard curves and calculated in relation to Gapdh/*GAPDH* as housekeeping gene. Expression levels are given as fold change of relative expression of a treated sample in comparison with the respective untreated control (=1).

### Mass-Spectrometric Analysis

Primary mouse lung fibroblasts isolated from S100A4^−/−^ mice were grown in RPMI-1640 supplemented with 10% fetal bovine serum, 100 U/ml penicillin, and 100 µg/ml streptomycin to about 80% confluency. Cell layers were treated with 1 µg/ml S100A4 for 5 or 15 min, or remained untreated. Proteins were prepared as previously described (30). In brief, total proteins from harvested cells were extracted by acetone/ethanol precipitation, and protein solutions diluted in 100 mM triethylammonium bicarbonate buffer (pH 8.2; TEAB) and 8 M urea. Proteins were reduced by 10 mM dithiothreitol at 60°C for 1 h and subsequently alkylated by 20 mM iodoacetic acid for 30 min. Samples were diluted by five volumes TEAB, received TPCK–trypsin at an enzyme-to-protein ratio of 1/25 and digested at 37°C for 16 h. For light, medium, and heavy dimethyl labeling, 300 µl of a mixture of CH_2_O, CD_2_O, and ^13^CD_2_O (4% each; Sigma-Aldrich, St. Louis, MO, USA) was added into 1 mg protein digest followed by 300 µl freshly prepared 600 mM NaBH_3_CN. After labeling, the three samples were mixed and enriched with Ti4^+^-IMAC microspheres (A kind gift from Prof. Mingliang Ye’s lab). Eluted phosphopeptides were analyzed by online SCX-RPLC–MS/MS using an LTQ Orbitrap XL mass spectrometer coupled with a quaternary surveyor MS pump (Thermo Fisher Scientific, San Jose, CA, USA). Full mass scan was acquired from 400 to 2,000 *m*/*z* (*R* = 6e5 at 400 *m*/*z*) with the target ion setting of 106. The 10 most intense ions were selected for fragmentation in the LTQ (30–32). The raw MS spectra were searched using MaxQuant version 1.3.0.5 (33) against the UniProt human database (12/11/2013), supplemented by frequently observed contaminants, and concatenated with reversed versions of all sequences. Trypsin was chosen with two missed cleavage sites. Phosphorylation (S, T, Y), oxidation (M), loss of ammonia and water were chosen for variable modifications; carbamidomethyl was chosen for fixed modifications. Triple dimethyl (28, 32, and 36) at N-termini and K were set for quantification. The maximum false-discovery rate was set to 1% for both the peptides and proteins, the minimum peptide length was 6. All the phosphorylation sites reported here were Class I sites with localization probability > 0.75 and ΔPTM score ≥ 5.

### Statistical Analysis

If not indicated otherwise, data are expressed as mean ± SEM, and combined raw data are shown. Differences between two groups were compared using Mann–Whitney (GraphPad Prism, La Jolla, CA, USA), and grouped comparison was evaluated by non-parametric ANOVA and subsequent Kruskal–Wallis (Kruskal–Wallis). Pearson test was used for analysis of correlations between two parameters. *p* Values < 0.05 were considered statistically significant.

## Results

### Extracellular S100A4 or S100A4^+^ Cells Are Increased in BALF of IPF Patients

**Figure 1:**
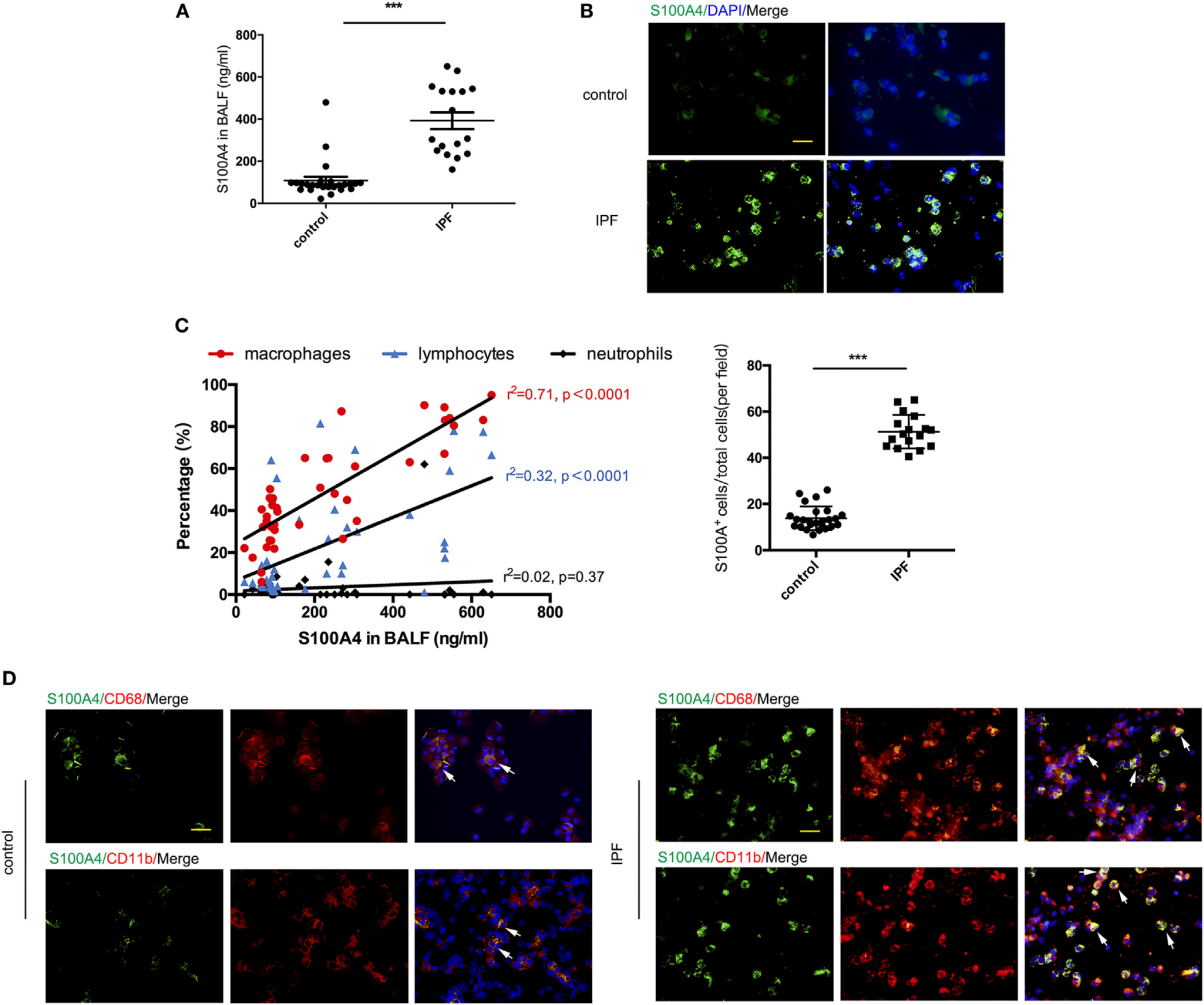
Extracellular S100A4 or S100A4^+^ cells are increased in bronchoalveolar lavage fluid (BALF) of idiopathic pulmonary fibrosis (IPF) patients. Soluble and cell-associated S100A4 was assessed from the BALF of patients with IPF (*n* = 17) or non-IPF lung disease (*n* = 25). **(A)** Levels of soluble S100A4 protein detected by enzyme-linked immunosorbent assay. Mean ± SEM, ***p < 0.001, Mann–Whitney. **(B)** S100A4 (green) in cells from BALF smears was visualized by immunofluorescence (upper panel). S100A4^+^ cells of total cells in each field were shown (lower panel), ***p < 0.001, Mann–Whitney. **(C)** S100A4 concentrations in BALF were correlated with percentages of macrophages, lymphocytes, or neutrophils as determined by cytology from H&E-stained BALF smears. Fits for each cell type indicated by the correlation coefficient “*r*” from Pearson test. **(D)** S100A4 (green) in combination with CD68 (red; upper panel) or CD11b (red; lower panel) in cells from BALF smears of IPF or non-IPF lung disease donors. **(B,D)** Representative images for each patient group, nuclei counterstained with 4′,6-diamidino-2-phenylindole (DAPI) (blue), scale bars, 25 µm.

First, we asked whether S100A4 expression levels in the lungs related to the extent of human pulmonary fibrosis. We studied non-cellular and cellular compartments of BALF from patients with IFP or non-fibrotic lung diseases. The level of extracellular S100A4 protein was about four times higher in the BALF of IPF group compared with the non-IPF control group, 392.7 ± 39.8 vs 108.4 ± 17.9 ng/ml (Figure 1A). In the BALF smears of suspended cells, half of the cells stained brightly for S100A4. By contrast, less than a quarter of the cells form non-IPF patients were S100A4^+^ and with dim cytoplasmic staining (Figure 1B). Based on the patients’ information in Table S1 in Supplementary Material, we correlated extracellular S100A4 protein levels to the relative abundance of immune-cell types in the patients’ BALF. Macrophages (*r* = 0.71), but not lymphocytes (*r* = 0.32) or neutrophils (*r* = 0.02) correlated with the level of soluble S100A4 (Figure 1C). So, next we double stained with the common macrophage marker CD68, it showed that in BALF smears a majority of S100A4^+^ cells were also CD68^+^ in IPF patients but a few S100A4^+^ cells emerged in non-IPF control patients (Figure 1D, upper panels). Similar staining patterns of S100A4 and CD11b as another macrophage marker strengthened the assumption that most of the S100A4^+^ cells within the BALF of IPF patients were macrophages (Figure 1D, lower panels).

The finding in human IPF showed increased S100A4 and suspended S100A4^+^ cells in BALF of IPF patients and pointed us to address the role of S100A4 for disease development.

### S100A4^+^CD11b^+^F4/80^+^ Cells Are Induced by Bleomycin Treatment in Lung and Correlate Directly With Pulmonary Fibrosis

**Figure 2:**
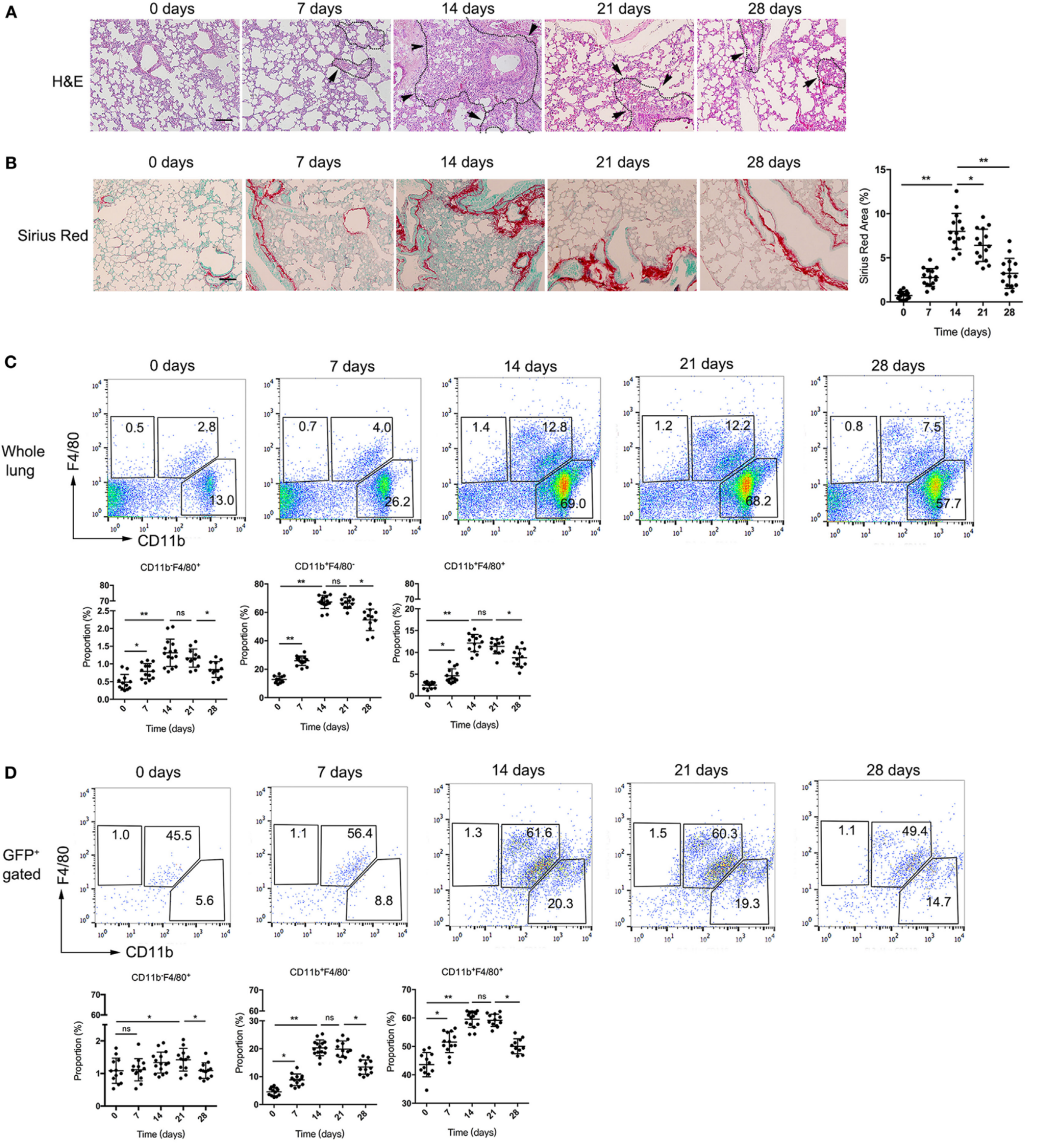

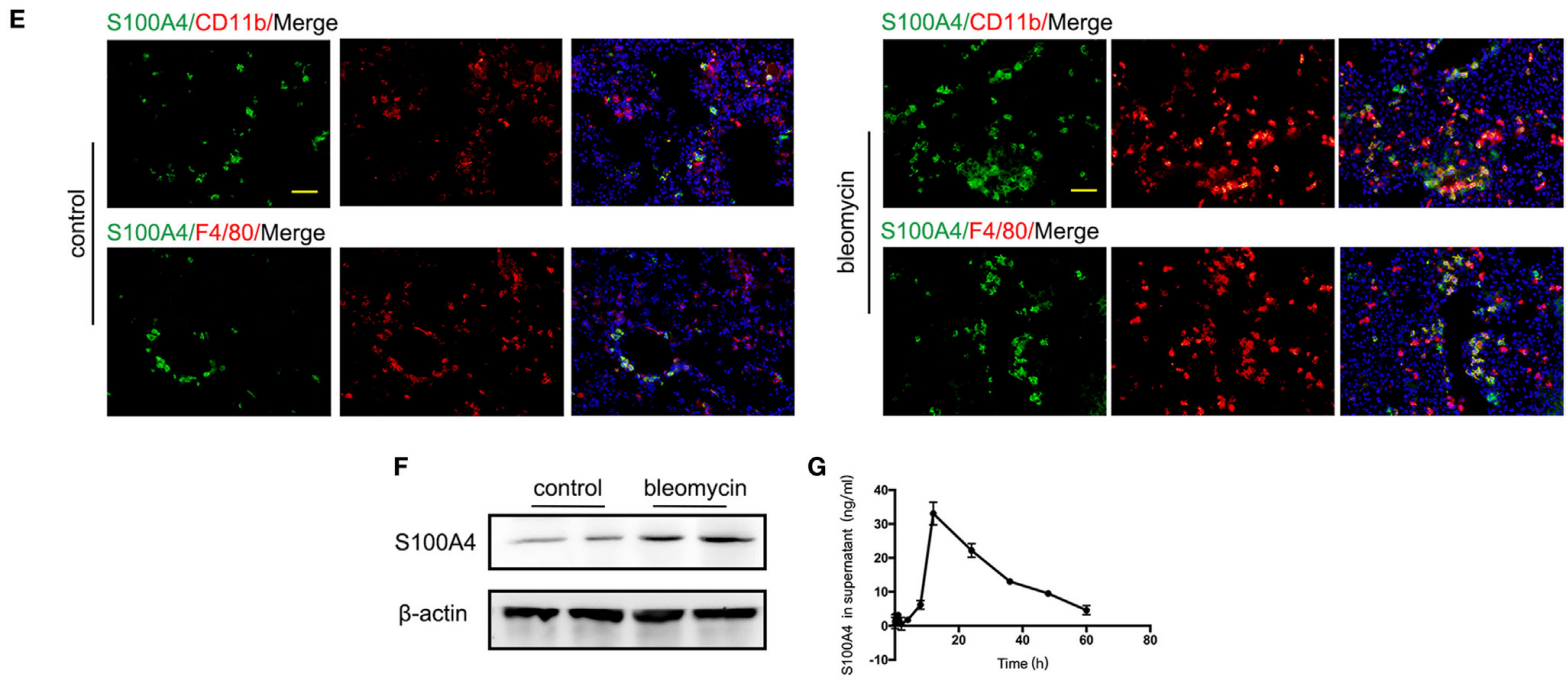
S100A4^+^CD11b^+^F4/80^+^ cells are induced by bleomycin treatment in lung and correlate directly with pulmonary fibrosis. **(A–D)** S100A4^+/+GFP^ or **(E,F)** WT mice were treated once with bleomycin to induce pulmonary fibrosis. **(A,B)** Lung tissues of bleomycin-treated S100A4^+/+GFP^ mice were stained by H&E and Sirius Red at the indicated time points. Age-matched untreated mice served as control (day 0). Representative images from three independent experiments. Arrows point to borders between immune-cell areas and alveolar cells; dotted lines highlight areas with severe immune-cell infiltration, scale bar 100 µm. Mean ± SEM from *n* = 5 mice per time point and experiment, *p < 0.05 and **p < 0.01, Kruskal–Wallis. **(C,D)** Whole lung-cell preparations stained for CD11b and F4/80 were analyzed in whole lung cells or in S100A4^+^ cells by flow cytometry. Representative dot plots from three independent experiments. Mean ± SEM from *n* = 4–5 per time point and experiment, *p < 0.05 and **p < 0.01, Kruskal–Wallis. **(E)** Lung tissues from WT mice 14 days after bleomycin treatment were stained for S100A4 (green) in combination with CD11b (red; upper panel) or F4/80 (red; lower panel). Representative images for three independent experiments with *n* = 5 per experiment, scale bar, 25 µm. **(F)** CD11b^+^F4/80^+^ macrophages (5 × 10^5^) freshly isolated from lung tissues of bleomycin-treated or untreated WT mice were subjected to western blot analysis for S100A4 (11.5 kDa). β-Actin (42 kDa) served as loading control. Blots of two mice per group representative for three independent *ex vivo* experiments. **(G)** Freshly isolated CD11b^+^F4/80^+^ macrophages (1 × 10^4^) from WT mice were cultured up to 60 h. S100A4 protein in the supernatant was detected over time by enzyme-linked immunosorbent assay. Data representative for three independent experiments, mean ± SD of *n* = 5 technical replicates per time point.

Addressing the source of soluble S100A4 in lung, we made use of *in vivo* mouse models of self-resolving pulmonary fibrosis that was induced by a single dose of bleomycin. Lung fibrosis was induced in S100A4^+/+GFP^ mice. We found immune-cell infiltration and alveolar-cell collapse reached a maximum after 14 days and subsequently declined over time (Figure 2A). Collagen deposition became visible after 7 days, increased until day 21, and was not completely resolved within the study period of 28 days (Figure 2B). Flow cytometry revealed percentage of CD11b^+^F4/80^+^ macrophages increased upon bleomycin treatment from 2.8% at day 0 to 12.8% at day 14 and subsequently decreased again (Figure 2C). Most of the S100A4^+^ cells were CD11b^+^F4/80^+^ macrophages (Figure 2D). Whereas below 5% of B cells as well as of CD4^+^, CD8^+^ T cells, and Ly6G^+^CD11b^+^ granulocytes were S100A4^+^ in the lung tissues at the peak time point of immune-cell infiltration 14 days after bleomycin application (Figures S1A,B in Supplementary Material). But the proportion of Ly6C^hi^CD11b^+^ monocytic myeloid cells in S100A4^+^ cells were elevated up to 20% (Figure S1B in Supplementary Material). Immunofluorescence of fibrotic lung tissue *in situ* confirmed these results. Large amount of S100A4^+^ cells in bleomycin-treated mice but not in the control mice expressed the macrophage markers CD11b or F4/80 (Figure 2E). Total cell extracts of CD11b^+^F4/80^+^ macrophages sorted from lung tissues showed more S100A4 protein in bleomycin-treated mice compared with control mice (Figure 2F; Figure S1C in Supplementary Material). S100A4 was also released by macrophages derived from bleomycin-treated fibrotic lungs (Figure 2G).

As S100A4 was once regarded a fibroblasts marker, we here studied its expression in mouse primary lung fibroblasts and fibrotic lung tissue. Mouse primary lung fibroblasts isolated from S100A4^+/+GFP^ transgenic mice and stained S100A4/α-SMA for immunofluorescence. It was found that S100A4 mainly located in nucleus, however, fibroblasts once activated by highly expressing α-SMA, S100A4 was deceased (Figure S2A in Supplementary Material). Accordingly, S100A4 did not show a clear colocalization with the myofibroblast marker ER-TR7 or α-SMA (Figures S2B,C in Supplementary Material).

These results suggested that during the progress of bleomycin-induced pulmonary fibrosis, a population of S100A4^+^CD11b^+^F4/80^+^ macrophages accumulated in lung tissue and correlated very well with the development of lung fibrosis.

### Extracellular S100A4 Promotes Lung Fibroblast Activation by Upregulating α-SMA Expression

As we showed that CD11b^+^F4/80^+^ macrophages in fibrotic lung tissue express high levels of S100A4, we asked for the mode of action of exogenous S100A4 during pulmonary fibrosis.

**Figure 3:**
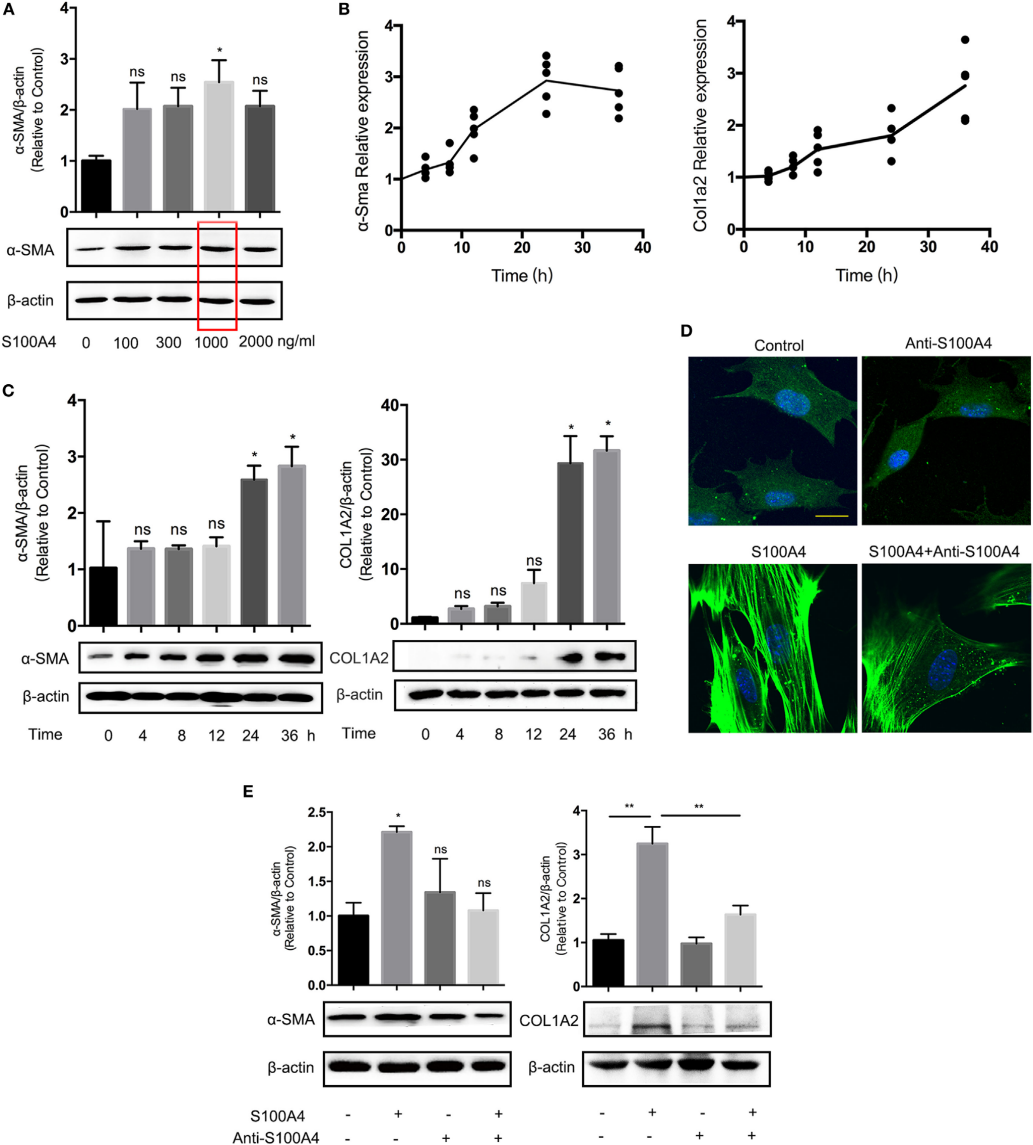
Extracellular S100A4 promotes lung fibroblast activation by upregulating α-SMA expression. Freshly prepared lung fibroblasts (1 × 10^5^) from untreated S100A4^−/−^ mice were treated *ex vivo* with recombinant S100A4. **(A)** After 24 h with increasing S100A4 concentrations, α-SMA (42 kDa) was evaluated by western blot analysis. β-Actin (42 kDa) served as a loading control. Three independent experiments with triplicate determinations. Representative images and mean ± SEM of α-SMA/β-actin ratios as quantified from individual band densities and relative α-SMA expression of treated cultures normalized to untreated control. Red box highlights S100A4 concentration used in all following experiments. *p < 0.05, Kruskal–Wallis compared with control cultures without exogenous S100A4. **(B,C)** Fibroblasts received recombinant S100A4 (1 µg/ml) for up to 36 h. **(B)** mRNA levels of α-Sma and Col1a2 were analyzed by real-time PCR in relation to Gapdh over time. Relative expression in treated cultures was normalized to the control without S100A4. Mean values of three independent experiments with *n* = 5 cultures per group and experiment. **(C)** α-SMA or COL1A2 (129 kDa) protein levels were determined by western blot analysis as described for panel **(A)**. Three independent experiments with triplicate determinations. Representative images and mean ± SEM, *p < 0.05, Kruskal–Wallis compared with control cultures without exogenous S100A4. **(D,E)** Fibroblasts were treated with S100A4 (1 µg/ml) for 24 h alone or in the presence of the neutralizing S100A4-specific antibody clone 3B11 (10 µg/ml). Cultures without S100A4 and/or anti-S100A4 served as controls. **(D)** Cells were stained for α-SMA (green) by immunofluorescence; nuclei (blue) counterstained with 4′,6-diamidino-2-phenylindole. Representative images from three independent experiments with *n* = 5 cultures per experiment. Scale bar 40 µm. **(E)** α-SMA and COL1A2 protein levels were analyzed by western blot analysis as described for panel **(C)**. Three independent experiments with triplicate determinations. Representative images and mean ± SEM, *p < 0.05 and **p < 0.01, Kruskal–Wallis.

If primary lung fibroblasts isolated from S100A4^−/−^ mice were treated with recombinant S100A4 in a dose-dependent manner, α-SMA protein was elevated by at least 1 µg/ml S100A4 (Figure 3A). The concentration was used for the following *in vitro* experiments. The mRNA levels of α-Sma and Col1a2 as markers for fibroblast activation were upregulated about threefold compared with the control group (Figure 3B). A peak of α-Sma expression emerged at 24 h, which was earlier than for Col1a2. It revealed different kinetics of the two related processes: early maximum fibroblast activation and ongoing collagen deposition. Consistently, α-SMA and COL1A2 protein levels were increased over time (Figure 3C). After treatment with recombinant S100A4, primary lung fibroblasts from S100A4^−/−^ mice showed a typical myofibroblast-like morphology with more pronounced spread patterns and high α-SMA expression (Figure 3D). Activation of the lung fibroblasts as determined by the morphology (Figure 3D) as well as by α-SMA and COL1A2 protein (Figure 3E) was abrogated if the S100A4 was preincubated with neutralizing anti-S100A4 antibody. These results indicated that exogenous S100A4 could directly activate lung fibroblasts.

### S1P Is Involved in S100A4-Induced Lung Fibroblast Activation

**Figure 4:**
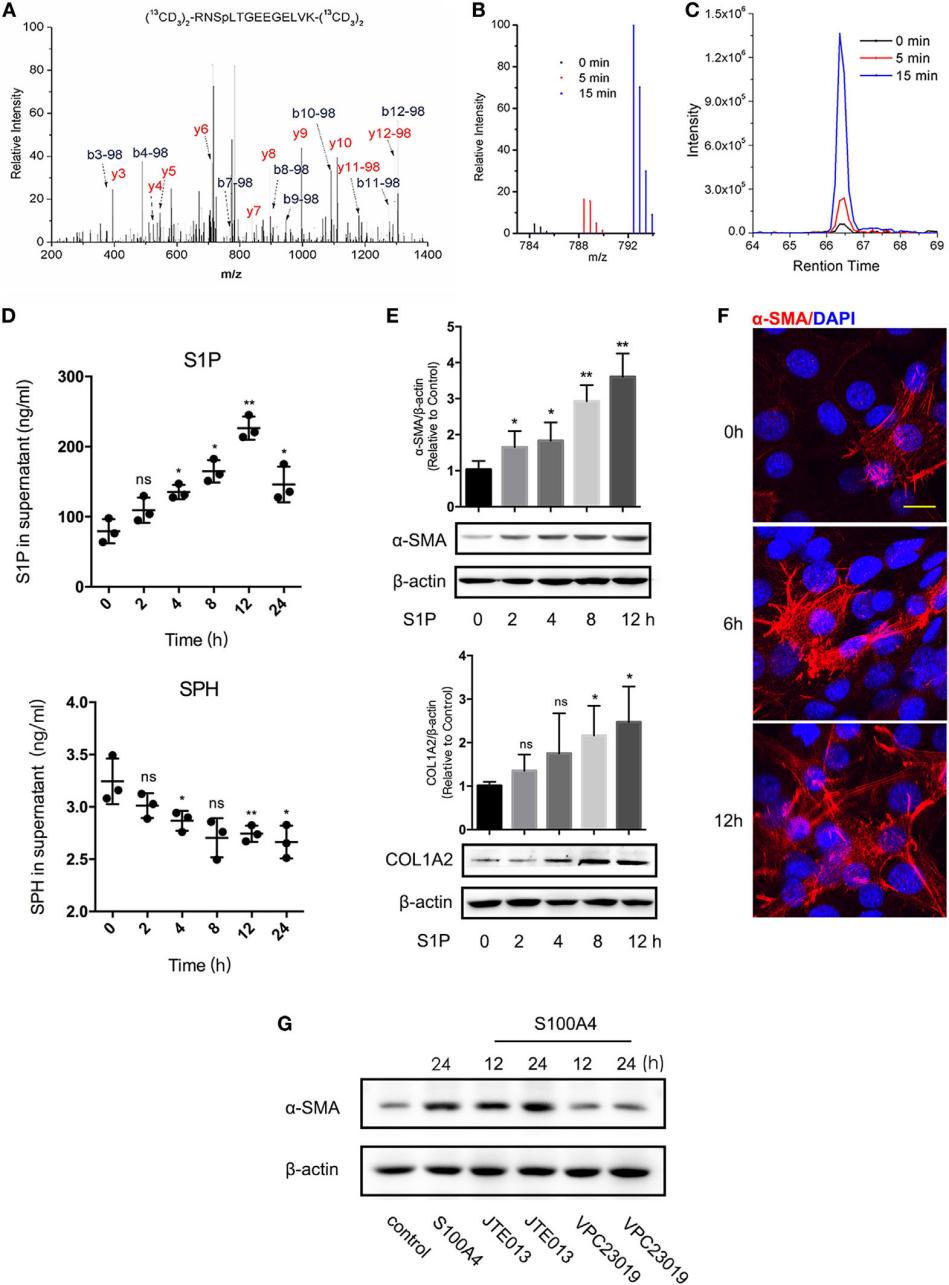
Sphingosine-1-phosphate (S1P) is essential in S100A4-mediated lung fibroblast activation. Fibroblasts were isolated from lung tissues of untreated S100A4^−/−^ mice. Fibroblasts were isolated from lung tissues of untreated S100A4^−/−^ mice. **(A–C)** Cells (1 × 10^8^) were cultured with recombinant S100A4 (1 µg/ml) for 5 (red) or 15 min (blue) or were left untreated (black). Total proteins were extracted, labeled, and subjected to MS/MS spectroscopy. The phosphopeptide RNSpLTGEEGELVK identified SGPP1 as different in the three treatment groups. **(A)** MS/MS spectra of the heavy dimethyl-labeled phosphopeptide. **(B)** MS spectrum of the light, medium, and heavy dimethyl-labeled phosphopeptide. **(C)** XIC peaks of the phosphopeptide abundance. **(D)** Fibroblasts were (1 × 10^5^) cultured for up to 24 h in the presence of recombinant S100A4 (1 µg/ml) or left untreated. Production of S1P (upper panel) and sphingosine (SPH) (lower panel) was determined from the culture supernatants by enzyme-linked immunosorbent assay. Three independent experiments with *n* = 5 per group and experiment. Mean ± SEM. **(E,F)** Fibroblasts (1 × 10^5^) were cultured for up to 12 h in the presence of S1P (20 µM). **(E)** Protein levels of α-SMA and COL1A2 were evaluated by western blot analysis as described for Figure 3C. Three independent experiments with triplicate determinations. Representative images and mean ± SEM, *p < 0.05 and **p < 0.01, Kruskal-Wallis compared with control cultures without S1P. **(F)** Cells were stained for α-SMA (red) by immunofluorescence; nuclei (blue) counterstained with 4′,6-diamidino-2-phenylindole (DAPI). Representative images from three independent experiments from *n* = 3 cultures per experiment, scale bar 25 µm. **(G)** Fibroblasts (1 × 10^5^) were treated with S100A4 combined with JTE013 (1 µM) or VPC23019 (10 µM) for up to 24 h. α-SMA or β-actin was shown by western blot analysis as described for Figure 3C. Representative images from two independent experiments with *n* = 3 cultures per experiment.

Next, we investigated molecular mechanisms involved in the activation of lung fibroblasts by S100A4. Quantitative phosphoproteomics of four sets of mice lung fibroblasts totally quantified 807, 860, 850, or 835 highly confident phosphorylation sites. Of these, 178 phosphorylation sites were highly confident in all four experiments (*p* < 0.05). We found 52 (5 min) and 53 sites (15 min) upregulated upon treatment with S100A4 compared with the untreated control; 49 sites at both time points (data not shown). The phosphopeptide RNSpLTGEEGELVK from SGPP1 was identified with high confidence, and the corresponding phosphorylation site S101 was upregulated by 4.1-fold (5 min) or 25.4-fold (15 min) after S100A4 treatment (Figures 4A–C). The evolutionarily conserved S101 position indicated it may carry an essential impact on the function of SGPP1 (Figure S3 in Supplementary Material).

We tested the hypothesis that the altered phosphorylation of S101 upon S100A4 treatment modified the enzymatic activity of SGPP1 in lung fibroblasts. Indeed, the concentration of the SGPP1 product SPH decreased while the concentration of the SGPP1 substrate S1P increased in a time-dependent manner after S100A4 treatment (Figure 4D). Sphingosine kinases (SPHK) directly induce S1P synthesis (34), and TGF-β mediates SPHK upregulation in mouse lung fibroblasts (35). We here treated freshly isolated lung fibroblasts with recombinant S100A4. SPHK1 mRNA and protein levels reached a maximum within 12 h that was already decreased after 24 h (Figures S4A,B in Supplementary Material, left panel). In the control with TGF-β, the signals for SPHK1 sustained longer and were overall stronger (Figures S4A,B in Supplementary Material, right panel).

We concluded that in addition to increased SPHK1, an S100A4-induced S101 phosphorylation inactivated the SGPP1 strongly contributed to an overall high S1P in the presence of S100A4.

Like S100A4, exogenous S1P induced the activation of lung fibroblasts as determined by upregulated α-SMA and COL1A2 expression (Figure 4E). Six hours after S1P stimulation, α-SMA was increased in some fibroblasts and after 12 h most lung fibroblasts showed an activated phenotype with a pronounced spreading morphology (Figure 4F). Emphasizing the functional connection between S1P and S100A4, we measured S1P receptor expression upon S100A4 stimulation. Of the five subtypes, S1P_1_, S1P_3_, and S1P_5_ increased after S100A4 treatment, S1P_2_ and S1P_4_ were downregulated (Figure S5 in Supplementary Material). Then, we used S1P_1_/S1P_3_ antagonist VPC23019 and S1P_2_ antagonist JTE013 to block S1P-related receptors. VPC23019 could abrogate S100A4-induced α-SMA upregulation, but JTE013 had no effects (Figure 4G). We assumed that with S100A4, S1P may activate downstream signals *via* S1P_1_/S1P_3_, but not through S1P_2_. ERK, p38, and AKT were not activated by S100A4 in our experiments with mouse primary lung fibroblasts (Figure S6 in Supplementary Material). These results indicated that S100A4 might activate lung fibroblasts by involving S1P signaling rather than *via* its canonical MARK or AKT signaling.

### Block of Extracellular S100A4 Abrogates Pulmonary Fibrosis and the Increased S1P *In Vivo*

**Figure 5:**
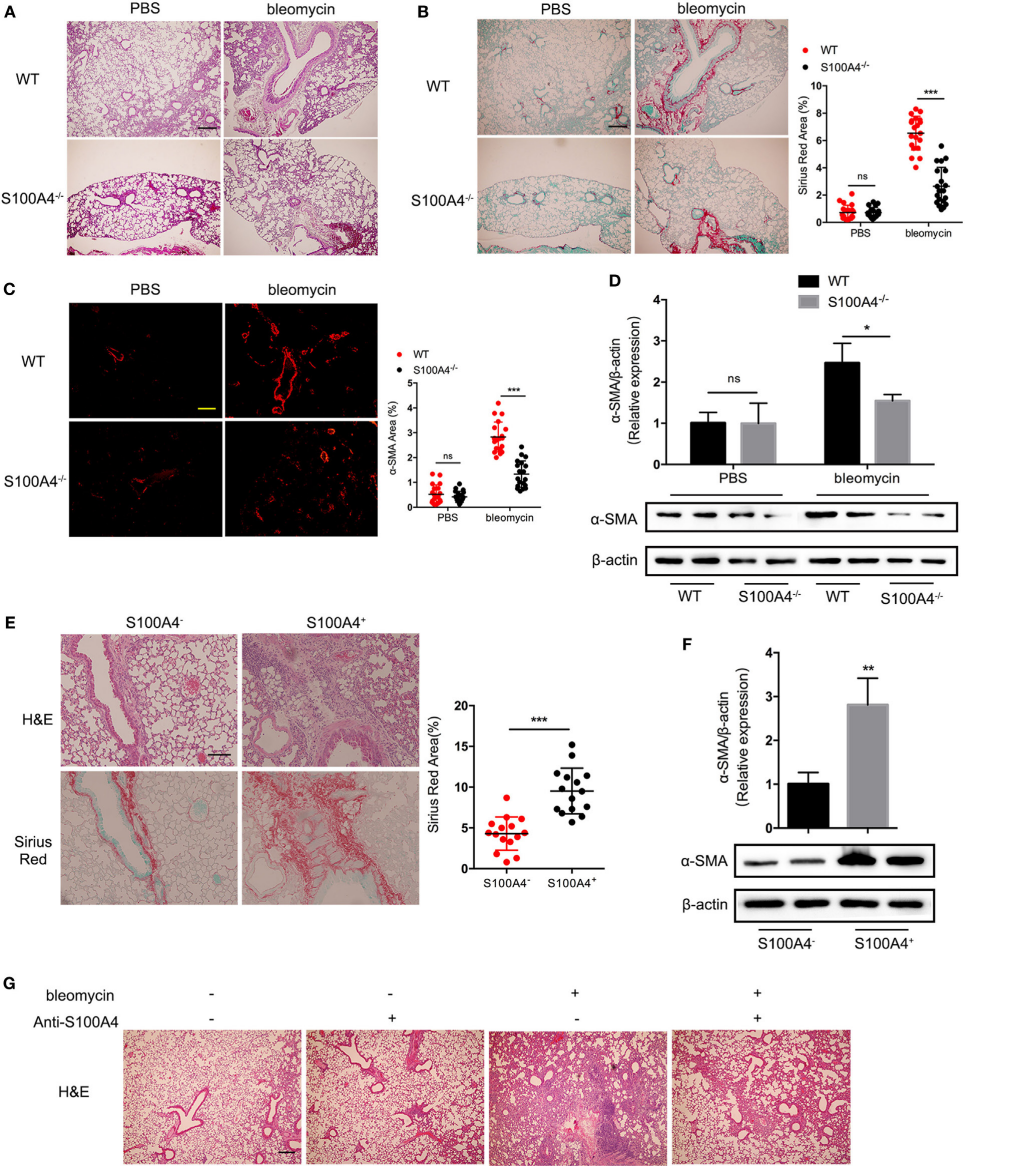

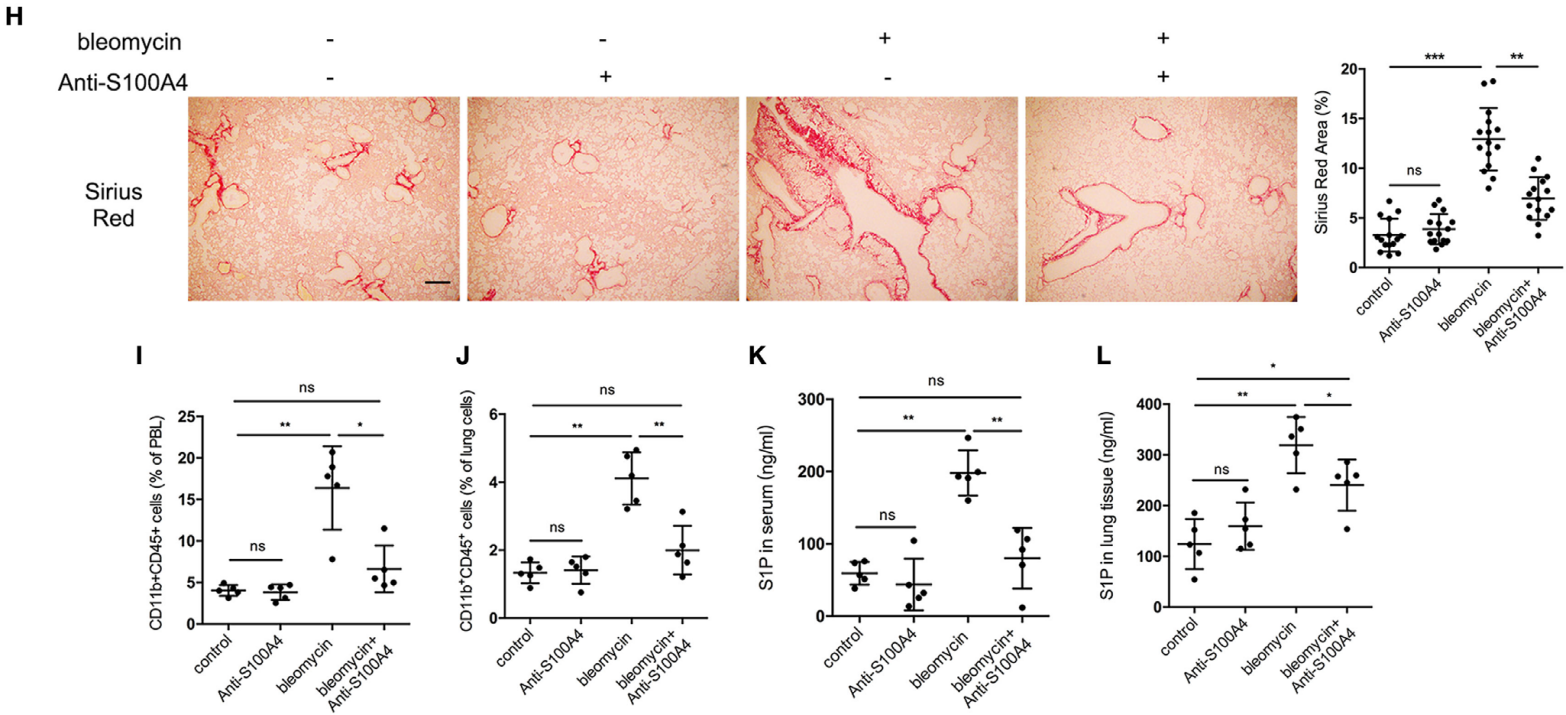
Block of extracellular S100A4 abrogates pulmonary fibrosis and the increased sphingosine-1-phosphate (S1P) *in vivo*. **(A–D)** WT and S100A4^−/−^ mice were treated once with or without bleomycin. Lung tissues were studied 14 days later. Three independent experiments with *n* = 5 mice per time point and experiment. **(A)** H&E staining for tissue integrity and immune-cell infiltration. **(B)** Sirius Red staining for collagen deposition. **(C)** α-SMA (red) stained by immunofluorescence. **(A–C)** Representative images, scale bar 100 µm. Mean ± SEM, ***p < 0.001, Mann–Whitney. **(D)** Protein levels of α-SMA were evaluated by western blot analysis as described for Figure 3C. Representative blots from two mice per group and mean ± SEM, *p < 0.05, Mann–Whitney. **(E,F)** S100A4^−/−^ mice received spleen-derived CD11b^+^ cells (2 × 10^6^) from S100A4^+/+GFP^ mice either positive or negative for S100A4 as sorted *via* the GFP reporter. Lung tissues were studied after 14 days. Three independent experiments with *n* = 5 mice per group and experiment. **(E)** H&E or Sirius Red staining for tissue integrity and immune-cell infiltration or collagen deposition. Representative images, scale bar 100 µm. Mean ± SEM, ***p < 0.001, by Mann–Whitney. **(F)** Protein levels of α-SMA were evaluated by western blot analysis as described for Figure 3C. Representative blots from two mice per group and mean ± SEM, *p < 0.05, Mann–Whitney. **(G–L)** WT mice were treated once with bleomycin. Starting at day 0, they received five repeated doses of anti-S100A4 (4 mg/kg) every third day. After 14 days, lung tissues and blood were studied. Three independent experiments with *n* = 5 mice per group and experiment. **(G)** H&E staining for tissue integrity and immune-cell infiltration. **(H)** Sirius Red staining for collagen deposition. Representative images, scale bar 100 µm. Mean ± SEM, **p < 0.01 and ***p < 0.001, Kruskal–Wallis. **(I)** PBL or **(J)** suspended lung cells stained for CD11b and CD45 were assessed by flow cytometry. S1P concentrations in panel **(K)** serum or **(L)** lung tissue were detected by enzyme-linked immunosorbent assay. **(I–L)** Mean ± SEM, *p < 0.05 and **p < 0.01, Kruskal–Wallis.

To further investigate whether absent S100A4 could attenuate pulmonary fibrosis *in vivo*, S100A4^−/−^ and WT mice were treated with bleomycin. Two weeks later, the lung tissue of WT mice was more severely infiltrated and destroyed compared with that of S100A4^−/−^ mice (Figure 5A). Collagen deposition in WT mice was nearly threefold higher than in the S100A4^−/−^ mice (Figure 5B). The expression of α-SMA was significantly lower in bleomycin-treated S100A4^−/−^ mice compared with their WT counterparts (Figures 5C,D). These results suggest that S100A4 deficiency was sufficient to abrogate fibroblast activation and attenuate pulmonary fibrosis *in vivo*. Establishing the connection to S100A4-producing macrophages, we adoptively transferred CD11b^+^S100A4^+^ or CD11b^+^S100A4^−^ cells to S100A4^−/−^ mice. Immune-cell infiltration and tissue damage were more severe in the mice that received S100A4^+^ cells compared with the group that received S100A4^−^ cells (Figure 5E, upper panel). Consistently, collagen deposition (Figure 5E, lower and right panels) and α-SMA expression were elevated significantly (Figure 5F). Further S100A4^−/−^ mice treated with both bleomycin in combination with exogenous S100A4 showed large amount of collagen deposition in the lung tissue compared with mice left untreated or treated with bleomycin only (Figure S7 in Supplementary Material), this addressed extracellular S100A4 pro-fibrotic function *in vivo*.

We next tested the potential of neutralizing S100A4 in preventing lung fibrosis *in vivo* (Figures 5G–L). The anti-S100A4 alone had no effect on the lung tissues of WT mice while bleomycin-induced pulmonary fibrosis with strong immune-cell infiltration and alveolar destruction as well as collagen deposition, simultaneous anti-S100A4 nicely prevented all signs of bleomycin-induced pulmonary fibrosis (Figures 5G,H). The proportions of CD45^+^CD11b^+^ monocytes in peripheral blood (Figure 5I; Figure S8A in Supplementary Material) and lung tissue (Figure 5J; Figure S8B in Supplementary Material) that were increased about threefold upon bleomycin administration and then reduced to baseline levels by anti-S100A4 treatment. Anti-S100A4 also normalized the bleomycin-induced S1P increase in serum (Figure 5K) and lung tissue that here contained blood-filled blood vessels (Figure 5L).

These findings strongly pointed to a therapeutic potential for blocking S100A4 in pulmonary fibrosis that also might be monitored by serum S1P levels.

### Extracellular S100A4 Functions Are Confirmed in Human Lung Fibroblasts

**Figure 6:**
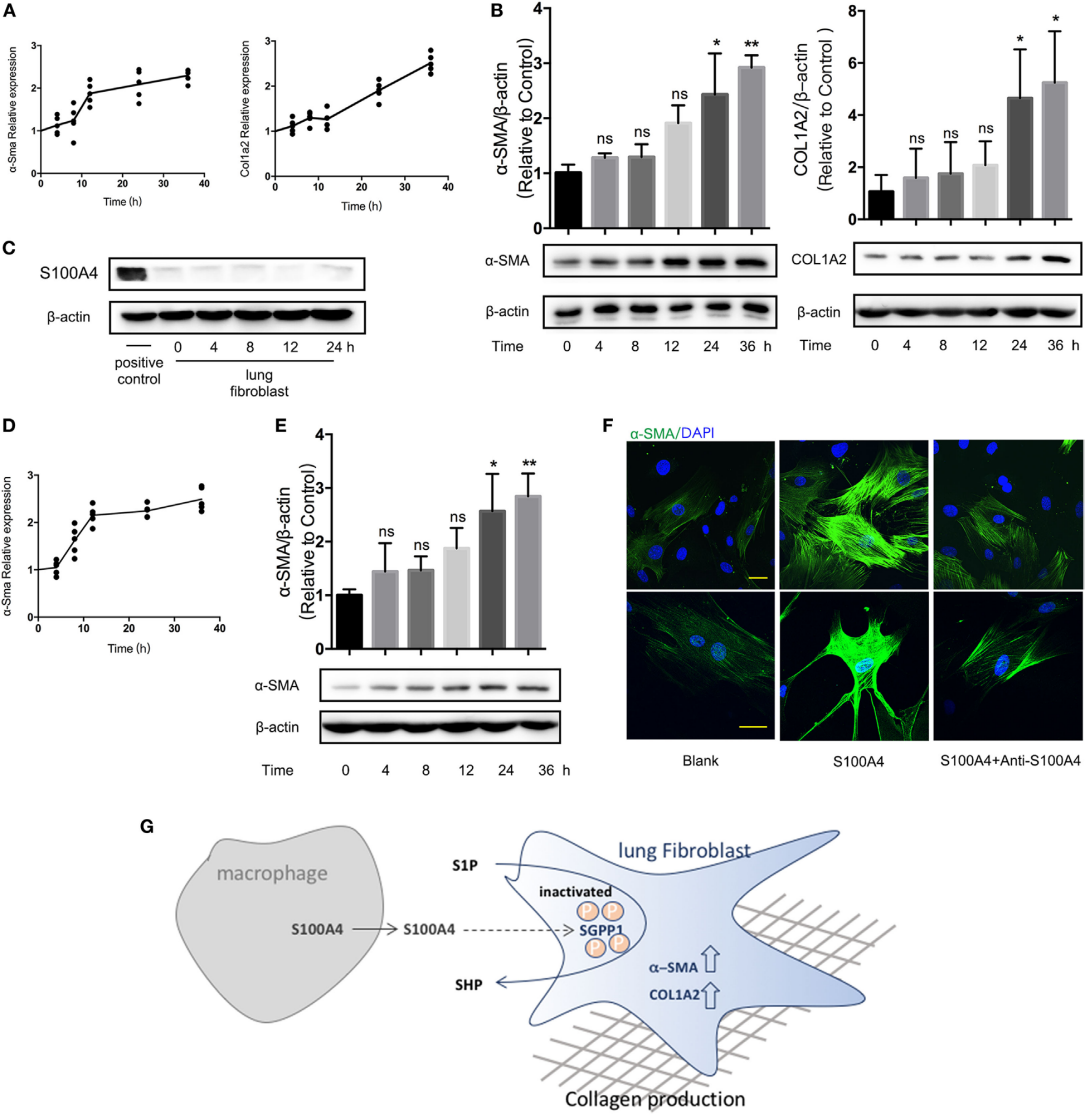
Extracellular S100A4 functions are confirmed in human lung fibroblasts. **(A,B)** Cells of the human lung fibroblast cell line MRC-5 were treated *in vitro* with recombinant S100A4 (1 µg/ml) for up to 36 h. Three independent cell-culture experiments with *n* = 5 cultures per group and experiment. **(A)** mRNA levels of *ASMA* and *COL1A2* were analyzed by real-time PCR in relation to *GAPDH*. Mean values of relative expression over time in treated cultures as normalized to the control without S100A4. **(B)** α-SMA and COL1A2 protein levels were determined by western blot analysis as described for Figure 3C. Representative blots and mean ± SEM, *p < 0.05 and **p < 0.01, Kruskal–Wallis. **(C–F)** Freshly isolated human primary lung fibroblasts were treated *ex vivo* with recombinant S100A4 (1 µg/ml) for up to 36 h. Three independent cell-culture experiments with *n* = 5 cultures per group and experiment. **(C)** S100A4 protein within the lung fibroblasts was detected by western blot analysis over time. RAW264.7 cells served as positive control. **(D,E)** mRNA and protein levels of *ASMA* were quantified as described for panels **(A,B)**. **(F)** Human lung fibroblasts treated with or without recombinant S100A4 and/or anti-S100A4 as indicated for 36 h were stained for α-SMA (green) by immunofluorescence. Representative images from two cultures per treatment group, scale bar, 25 µm. **(G)** Schematic summary for a role of S100A4 released by macrophages in activating of lung fibroblasts through an S1P-dependent upregulation of α-SMA.

We next wanted to confirm the crucial role of S100A4 for the activation and collagen production of lung fibroblasts in human cells. The expression of *ASMA* and *COL1A2* mRNA (Figure 6A) or α-SMA and COLA2 protein (Figure 6B) increased significantly if cells of the human lung fibroblast cell line MRC-5 were exposed to exogenous S100A4. Primary human lung fibroblasts expressed overall very low levels of S100A4. Excluding an autocrine induction, it was not upregulated by recombinant S100A4 (Figure 6C). Upon S100A4 administration, *ASMA* mRNA was to 2.5-fold increased within 36 h (Figure 6D). The α-SMA protein was also elevated over time (Figure 6E). Comparable to the findings with primary mouse fibroblasts (see Figure 3D), neutralizing S100A4 function with anti-S100A4 antibody lowered the density of α-SMA (Figure 6F). These observations verified the causal relation of extracellular S100A4 to the fibrotic process in human pulmonary fibrosis.

## Discussion

Pulmonary fibrosis is a significant health concern and a better understanding of underlying mechanisms may help to develop novel therapeutic strategies. We show here in a model of bleomycin-induced pulmonary fibrosis that S100A4 from CD11b^+^F4/80^+^ macrophages activate fibroblasts in a process that involves the modulation of S1P levels (Figure 6G). This was proven by the findings that S100A4 deficiency *in vivo* attenuated lung fibrosis and neutralizing exogenous S100A4 by anti-S100A4 has the potential to prevent the fibrosis. Since the S100A4 protein in the BALF of IPF patients was increased, our results provide a basis for improving the diagnosis and supply new potential targets of pulmonary fibrosis therapy.

S100A4 has been first identified and was applied as a specific marker of fibroblasts (36). Many studies revealed S100A4 in tissue fibrosis, such as in liver, kidney, or heart, but the specific role of S100A4 is different in each entity (37). Intricacies of the expression and the manifold biological functions of S100A4 were figured out only in recent years. In cardiac fibrosis about half of the S100A4^+^ cells are of hematopoietic origin and many endothelial cells also express S100A4 (38). S100A4 produced by an inflammatory subpopulation of macrophages in the liver (26) promotes liver fibrosis *via* activation of hepatic stellate cells (18). A subpopulation of macrophages was the major source for S100A4 in our model of bleomycin-induced pulmonary fibrosis and in human IPF. Although we cannot completely exclude other cell types, we conclude that S100A4^+^ macrophages were most important during the inflammatory phase since collagen-producing myofibroblasts showed low S100A4 expression. Prior reports indicated that few cells stain positive for both S100A4 and α-SMA but morphological analysis attributed S100A4 mostly to fibroblasts (23). Mesenchymal progenitor cells in IPF also express S100A4 but loose it upon differentiation to α-SMA^+^ myofibroblasts (24). In this case, S100A4^+^ cells were not classical collagen-producing myofibroblasts. Abundant myofibroblasts in pulmonary fibrosis after irradiation originate from the bone marrow (39), and a large proportion of bone marrow-derived collagen-producing cells do not express α-SMA (40). The function of S100A4 from macrophages we show here is an important addition to understand cellular mechanisms of pulmonary fibrosis. S100A4 plays a critical role in the fibrotic tissue remodeling in lung, liver, and kidney. Although parts of recruited pathways seem similar in different tissues, diverse organ functions result in different outcomes that need to be understood further. S1P is instrumental in regulating many biological processes and also promote α-SMA expression in fibroblasts (41). This includes the activation of lung fibroblasts and the development of lung fibrosis (42, 43). SGPP1 mainly located in the endoplasmic reticulum reversibly dephosphorylates S1P to SPH and therefore represents the crucial factor for the balance of SPH and S1P (44). Concerted enzymatic activity of SPHK, SGPP1, and sphingosine-1-phosphate lyase regulate S1P synthesis and degradation (34). On the one hand, S100A4 induced a transient increase in SPHK mRNA and protein levels that were less pronounced than the effects TGF-β used as a positive control. On the other hand, S100A4 elevated the SGPP1 phosphorylation up to 25-fold. We therefore conclude that both two options contribute to a reduced dephosphorylation of S1P within the lung fibroblasts, hence were responsible for the overall S1P increase in response to exogenous S100A4. With macrophages as an important source of S100A4, this provides a new insight to the intricacies of fibrosis-related metabolic changes. Whether the interaction of S1P with its receptors by inhibitors like VPC23019 might be used clinically remain to be elucidated.

Another strategy could use S100A4 directly as a target for clinical intervention in IPF. Previous studies mainly focus on intracellular S100A4 in lung fibroblasts (23, 45), rendering it a difficult target to block by large biologicals like antibodies. Our previous work showed that a fusogenic liposome can deliver anti-S100A4 into the cytoplasm in a fusion-dependent manner bypassing the cellular endocytosis and avoid the inefficient escape in breast cancer (46).

We here did not specifically ask which subset of the S100A4^+^ macrophages belong to? Their association to fibroblast activation and tissue repair (18) rather point to a regulatory phenotype of these macrophages. This interesting topic, of course, requires further investigation. Our study here revealed large amounts of extracellular S100A4 that derived from macrophages during inherent inflammation as a new target for antibody-based therapies in IPF. Many questions concerning the impact of neutralizing exogenous S100A4 in IPF are pending. Finally, signaling of ERK, p38, or AKT that were found to be activated in pulmonary fibrosis (47–49), but they were excluded in our experiments may due to various cytokine action mode, so the details of S100A4 receptor and of molecular mechanisms for the connection of S100A4 with the enzymatic conversion of S1P are topic for future studies.

What are the main obstacles resulting from the ubiquitous nature as well as the intracellular and extracellular functions of S100A4? Intracellular S100A4 in the pathologic mesenchymal progenitor cells of IPF localizes to the nucleus and promotes p53 degradation (24). We showed that macrophages can express and release S100A4 that paracrinely induces fibroblast activation. Whether and how therapeutically inhibiting S100A4 disrupts the physiological functions of S100A4 is also still uncertain. In our S100A4-neutralizing experiments in the mouse model, local differences between normal pathological S100A4 levels were big enough to elicit different effects of the antibody in the treatment groups. Although they clearly prove the therapeutic potential, optimized targeted drug-delivery approaches might provide a more suitable treatment option. Nano-materials or respirable S100A4 antibody/microRNA should be considered.

Overall, effective therapies and diagnosis for pulmonary fibrosis are still limited to date. Thus, innovative anti-fibrotic therapies attenuating or resolving fibrosis are urgently needed. Our study suggests S100A4 as diagnostic marker for IPF. It also provides clear evidence that blocking S100A4 might be beneficial for treating pulmonary fibrosis in the future.

## Ethics Statement

Human subjects: this study was carried out in accordance with the recommendations of Standard Operating Procedure and was approved by the ethics committee at Peking University People’s Hospital. The protocol was approved by the ethics committee at Peking University People’s Hospital. All subjects gave written informed consent in accordance with the Declaration of Beijing. Animal subjects: this study was carried out in accordance with the recommendations of Standard Operating Procedure, Institutional Laboratory Animal Care and Use Committee, Chinese Academy of Sciences. The protocol was approved by the Institutional Laboratory Animal Care and Use Committee.

## Author Contributions

ZQ and ZG conceived the project. YL performed animal experiments, immunohistological staining, and western blot analysis. YB carried out mass-spectrometric analysis. JB and ZG provided human species samples and gave suggestions on animal experiments. KS and SL participated in partial animal studies and carried out real-time PCR. PW designed partial experiments and revised the manuscript. YL, YB, and ZQ analyzed the data and drafted the manuscript. YL, UE, ZL, and ZQ finalized the paper.

## Conflict of Interest Statement

The authors declare that the research was conducted in the absence of any commercial or financial relationships that could be construed as a potential conflict of interest. The reviewer SH and handling Editor declared their shared affiliation.
