# Supplementary material for: S100A4+ Macrophages Are Necessary for Pulmonary Fibrosis by Activating Lung Fibroblasts
Source: Front Immunol. 2018 Aug 6;9:1776. doi: 10.3389/fimmu.2018.01776 (PMC6088238; doi:10.3389/fimmu.2018.01776)
Supplement: Supplementary file 1 [file data_sheet_1.PDF]

Supplementary Data

Supplementary Tables

Supplementary Table 1: Characteristics of patients in BALF study group

Parameter		IPF	Non-IPF lung disease
n		17	25
Sex	Male (n)	13	17
	Female (n)	4	8
Age in years (mean, range)		71.8, 44-88	60.8, 28-82
Immune cell types in % (mean ± SEM)			
	Monocytes/macrophages	63.2 ± 5.1	37.8 ± 4.0
	Lymphocytes	42.5 ± 5.9	10.6 ± 3.0
	Neutrophils	1.56 ± 0.9	4.56 ± 2.4
	Cell # x10^5/ml (mean ± SEM)	3.41 ± 0.13	4.66 ± 0.12

Supplementary Table 2: Primary antibodies used in flow cytometry of murine cells

Antigen	Clone/order #	Fluorochrome	Final dilution	Purchaser
B220	RA3-B62	APC	1:200	Biolegend, San Diego, CA
CD4	RM4-5	PE	1:250	Biolegend, San Diego, CA
CD8	53-6.7	PE	1:250	Biolegend, San Diego, CA
CD11b	M1/70	PERCP-cy5.5	1:200	Biolegend, San Diego, CA
F4/80	BM8	APC	1:200	Biolegend, San Diego, CA
Ly6C	HK1.4	PE	1:250	Biolegend, San Diego, CA
Ly6G	1A8	APC	1:200	Biolegend, San Diego, CA

Supplementary Table 3: Primary antibodies used in immunohistology or western-blot analysis with human or murine cells or tissues

Target species	Antigen	Clone/ order #	Final dilution	Purchaser
Human	CD11b	M1/70	1:200	BD Biosciences, San Diego, CA
	CD68	KP1	1:200	Abcam, Cambridge, UK
	S100A4	ab27957	1:200	Abcam, Cambridge, UK
Murine	α-SMA	ab5694	1:200	Abcam, Cambridge, UK
	β-actin	6G3	1:1500	Sungenebiotech, Tianjin China
	AKT	40D4	1:1000	Cell Signaling Technology, Boston, MA
	CD11b	M1/70	1:200	BD Biosciences, San Diego, CA
	COL1A2	ab96723	1:100	Abcam, Cambridge, UK
	ERK	3A7	1:1000	Cell Signaling Technology
	ER-TR7	ab51824	1:200	Abcam, Cambridge, UK
	F4/80	BM8	1:200	Biolegend, San Diego, CA
	P38	L53F8	1:1000	Cell Signaling Technology, Boston, MA
	p-AKT	D9E	1:1000	Cell Signaling Technology, Boston, MA

p-ERK	D13.14.4E	1:1000	Cell Signaling Technology, Boston,
p-P38	D3F9	1:1000	MA Cell Signaling Technology,
S100A4	ab27957	1:200	Boston, MA Abcam, Cambridge, UK
SPHK1	ab71700	1:1000	Abcam, Cambridge, UK

Supplementary Figures

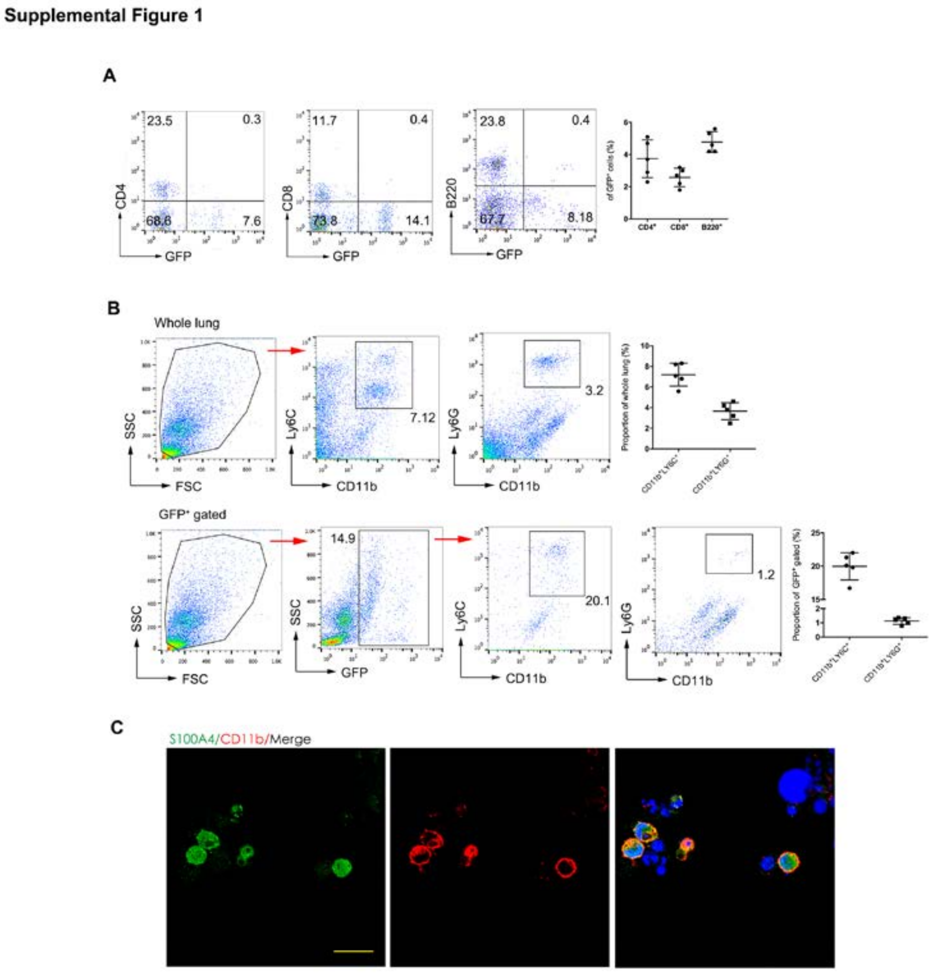

Supplementary Fig. 1: Low expression of S100A4 by lymphocytes and granulocytes. (A-C) S100A4$^{+/+GFP}$ mice were treated once with bleomycin. Three independent experiments with n=5 per group and experiment. Representative dot plots and Mean ± SEM. (A) After 14 days, whole lung cells stained for CD4, CD8 and B220 were assessed by flow cytometry. Percentages of CD4$^+$, CD8$^+$ or B220$^+$ lymphocytes within the S100A4$^+$ cell population as determined by the GFP reporter are given. (B) cells stained for CD11b, Ly6C and Ly6G were analyzed for percentages of CD11b$^+$Ly6C$^+$ and CD11b$^+$Ly6G$^+$ cells in whole lung cells (upper panel) or in S100A4$^+$ cells as gated by the GFP reporter (lower panel). (C) CD11b$^+$F4/80$^+$ macrophages were isolated from bleomycin treated lung tissues and stained by S100A4 (green) and CD11b (red) by immune-cytology. Nuclei (blue) were counterstained with DAPI. Representative images from three independent experiments with n=5 mice, scale bar 30

μm.

Supplemental Figure 2

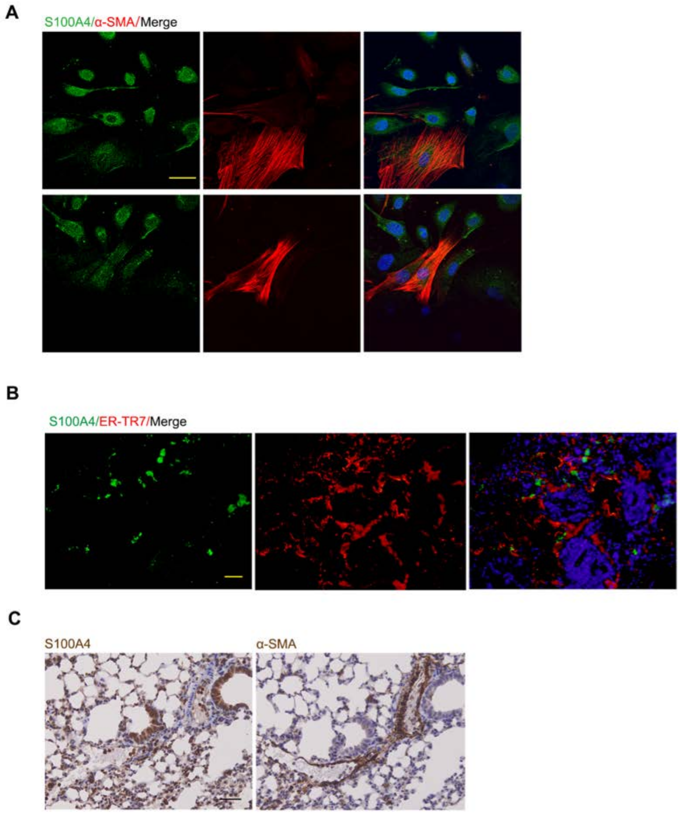

Supplementary Fig. 2: Lung myofibroblasts in the mouse models express low amounts of S100A4. (A) Primary lung fibroblasts from S100A4$^{+/+GFP}$ mice were isolated. Cells were stained for S100A4 (green) and α-SMA (red). scale bar 40 μm. (B, C) WT mice were treated once with bleomycin. (B) Immunofluorescent double stain for S100A4 (green) and ER-TR7 (red), scale bar 25 μm. (C) Immunohistochemistry (brown) for S100A4 (left panel) or α-SMA (right panel) in adjacent sections. Nuclei (blue) were counterstained with hematoxylin. Scale bar, 25 μm. (A-C) Representative images for three independent experiments with n=5 per experiment.

Supplemental Figure 3

```
                               .    :    ******.* *:   : *  *: *
  sp|Q9JI99|SGPP1_MOUSE EPGPTGPRRAGSQRRNSLTGEEGELVKVSNLPLYYL
  sp|Q9BX95|SGPP1_HUMAN ELGPASPRRAGALRRNSLTGEEGQLARVSNWPLYCL
tr|H2Q8G0|H2Q8G0_PANTR ELGPASPRRAGGLRRNSLTGEEGQLARVSNWPLYCL
tr|K9J0Z4|K9J0Z4_DESRO DLGPASPRRAGALRRNSLTGEEGELAYVSNWPLYYL
tr|J9NXT3|J9NXT3_CANLF ELGPASPRRVGALRRNSLTDEEGELAHVSNWPLYYL
    tr|I3L5T1|I3L5T1_PIG KLGPASPRRAGALRRNSLTGEEGQLAHVSNWPLYYL
tr|G3SNB3|G3SNB3_LOXAF ELGPAPPRRAGALRRNSLTGEEGQLAQVNNWPLYYL
tr|W5QJ25|W5QJ25_SHEEP EVGPASPRRAGLLRRNSLTGEEGQLAHVSNWPLHYL
tr|F6V798|F6V798_XENTR AHPALPQDKKKPQRRNSLTGEVGQEFLIRNRFLYYL
```

Supplementary Fig. 3: The phosphorylation site S101 of SGPP1 is highly conserved within vertebrae. Peptide sequences containing around the S101 of SGPP1 from *Mus musculus*, *Homo sapiens*, *Pan troglodytes*, *Desmodus rotundus*, *Canis lupus*

familiaris, Sus scrofa, Loxodonta africana, Ovis aries and *Xenopus tropicalis*. Multiple sequence alignment was performed by Clustal X and the phosphorylation site S101 is highlighted in grey. Data sources are indicated in the left column.

Supplemental Figure 4

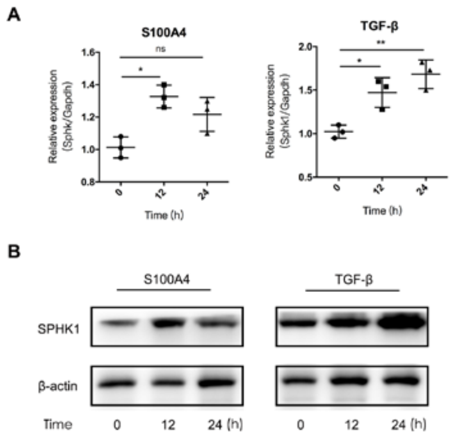

Supplementary Fig. 4: Treatment with S100A4 increases SPHK1 expression in primary lung fibroblasts. Lung fibroblasts were isolated from S100A4$^{-/-}$ mice. (A) Fibroblasts received extracellular S100A4 (1 µg/ml) or TGF-β (5ng/ml) as control for up to 24 h. mRNA levels of Sphk1 were analyzed by real-time PCR in relation to Gapdh. Relative expression in treated cultures was normalized to the control without S100A4 or TGF-β. Mean ± SEM of three independent experiments. $*p < 0.05$, $**p < 0.01$, Kruskal-Wallis. (B) Cells were treated as described in (A) before SPHK1 (48 kDa) and β-actin were detected by western-blot analysis. Representative images from two independent experiments with n=3 per cultures per expression.

Supplemental Figure 5

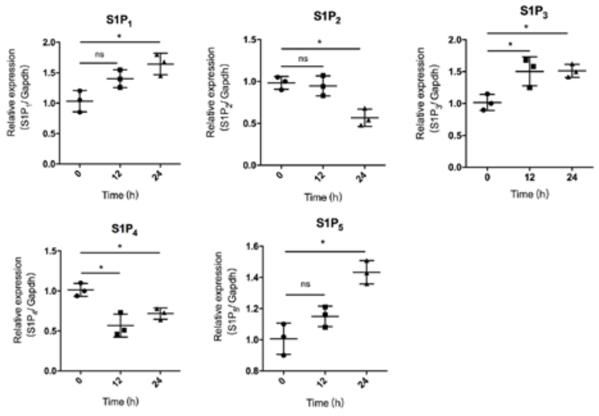

Supplementary Fig. 5: Exogenous S100A4 modulates S1P-receptors subtype expression in primary lung fibroblasts. Lung fibroblasts were isolated from S100A4$^{-/-}$ mice were treated with S100A4 (1 µg/ml) for up to 24 h. mRNA levels of $S1P_{1-5}$ were analyzed by real-time PCR in relation to Gapdh. Relative expression in treated cultures was normalized to the control without S100A4. Mean ± SEM of three independent

experiments. *p＜0.05, Kruskal-Wallis.

Supplemental Figure 6

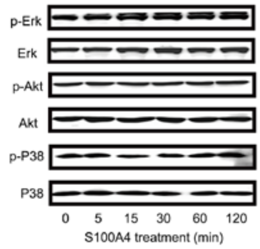

Supplementary Fig. 6: MAPK or AKT signal pathways are not activated in mouse fibroblasts after treatment with S100A4. S100A4$^{-/-}$ lung fibroblasts ($1x10^5$) were treated with recombinant S100A4 (1 µg/ml) up to 120 min. Activation of p-ERK (42/44 kDa), p-AKT (56 kDa), p-P38 (38 kDa) were detected by western-blot analysis. Representative images for three independent experiments with n=3 cultures per experiment.

Supplemental Figure 7

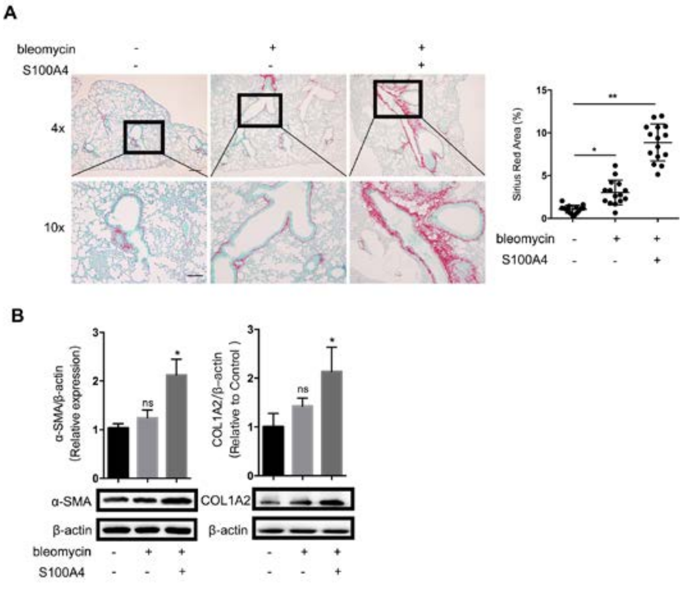

Supplementary Fig. 7: Exogenous S100A4 aggravates pulmonary fibrosis in S100A4$^{-/-}$ mice. S100A4$^{-/-}$ mice were treated once with bleomycin to induce pulmonary fibrosis. (A) Sirius Red staining for collagen deposition in lung tissues of S100A4$^{-/-}$ mice 14 days after bleomycin treatment with or without simultaneous injection of recombinant S100A4 (5 µg/mouse). Representative images from three independent experiments, scale bar 200 µm (upper)/100 µm (lower). Mean ± SEM from n=5 mice

per group. *p＜0.05, **p＜0.01, Kruskal-Wallis. (B) α-SMA and COL1A2 protein levels in lung tissues were evaluated by western-blot analysis as described for Figure 3C. Three independent experiments with triplicate determinations. Representative images and Mean ± SEM, *p＜0.05, Kruskal-Wallis compared to control cultures without bleomycin and exogenous S100A4.

Supplemental Figure 8

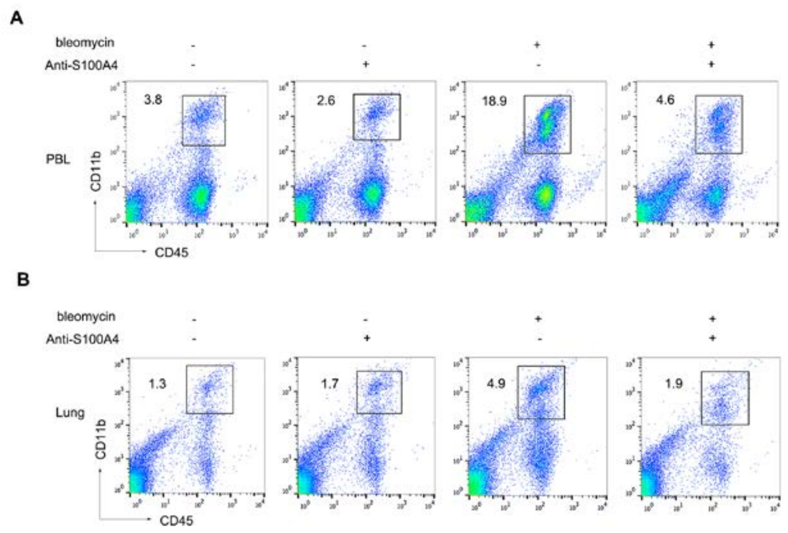

Supplementary Fig. 8: Anti-S100A4 treatment reduces CD45^{+}CD11b^{+} monocytic cells in blood and lung tissue *in vivo.* WT mice were treated with bleomycin in the presence or absence of anti-S100A4 as indicated. After 14 days, (A) PBL and (B) whole lung cells stained for CD45 and CD11b were analyzed by flow cytometry. Representative plots for three independent experiments with n=5 mice per group and experiment.
